# Perinatal Arterial Ischemic Stroke Is Associated to Materno-Fetal Immune Activation and Intracranial Arteritis

**DOI:** 10.3390/ijms17121980

**Published:** 2016-11-25

**Authors:** Clémence Guiraut, Nicole Cauchon, Martin Lepage, Guillaume Sébire

**Affiliations:** 1 Département de Pédiatrie, Université de Sherbrooke, Sherbrooke, QC J1H 5N4, Canada; clemence.guiraut@usherbrooke.ca; 2 Département de Médecine Nucléaire et Radiobiologie, Université de Sherbrooke, Sherbrooke, QC J1H 5N4, Canada; nicole.cauchon@usherbrooke.ca (N.C.); martin.lepage@usherbrooke.ca (M.L.); 3 Child Neurology Division, Department of Pediatrics, McGill University, Montréal, QC H4A 3J1, Canada

**Keywords:** gestational inflammation, vasculitis, perinatal arterial ischemic stroke, lipopolysaccharide

## Abstract

The medium-size intra-cranial arteries arising from the carotid bifurcation are prone to perinatal arterial ischemic strokes (PAIS). PAIS’ physiopathology needs to be better understood to develop preventive and therapeutic interventions that are currently missing. We hypothesized that materno-fetal inflammation leads to a vasculitis affecting selectively the carotidian tree and promoting a focal thrombosis and subsequent stroke. Dams were injected with saline or lipopolysaccharide (LPS) from *Escherichia coli*. A prothrombotic stress was applied on LPS-exposed vs. saline (S)-exposed middle cerebral arteries (MCA). Immunolabeling detected the inflammatory markers of interest. In S-exposed newborn pups, a constitutive higher density of macrophages combined to higher expressions of tumor necrosis factor-α (TNF-α), and interleukin 1β (IL-1β) was observed within the wall of intra- vs. extra-cranial cervicocephalic arteries. LPS-induced maternal and placental inflammatory responses mediated by IL-1β, TNF-α and monocyte chemotactic protein 1 (MCP-1) were associated with: (*i*) increased density of pro-inflammatory macrophages (M1 phenotype); and (*ii*) pro-inflammatory orientation of the IL-1 system (IL-1β/IL-1 receptor antagonist (IL-1Ra) ratio) within the wall of LPS-, vs. S-exposed, intra-cranial arteries susceptible to PAIS. LPS plus photothrombosis, but not sole photothrombosis, triggered ischemic strokes and subsequent motor impairments. Based on these preclinical results, the combination of pro-thrombotic stress and selective intra-cranial arteritis arising from end gestational maternal immune activation seem to play a role in the pathophysiology of human PAIS.

## 1. Introduction

Perinatal arterial ischemic stroke (PAIS) is the most frequent form of pediatric stroke [1,2,3]. It affects one in 2500 newborns [4]. During our lifetime, the day of birth is the one we are most vulnerable to stroke’s occurrence. PAIS leads to severe neurobehavioral morbidities such as hemiplegic cerebral palsy (CP), cognitive and learning impairments, or both [1]. A quarter of all CP occurrences derive from PAIS [5]. The causal pathway of PAIS needs to be better understood to implement preventive and therapeutic treatments that are currently inexistent [6]. For unknown reasons, almost all PAIS occur in the territories of intra-cranial arteries developing from the carotidian tree [7]—namely, the distal part of the intra-cranial internal carotid artery (icICA) and the proximal parts of anterior cerebral artery (ACA), middle cerebral artery (MCA), or posterior cerebral artery (PCA)—while basilar artery (BA) and extra-cranial (ec) arteries are not affected [3,4,8,9,10,11]. The most classic pathophysiological hypothesis postulates that the arterial occlusion might be due to emboli from the placenta or the umbilical cord reaching the brain through the fetal circulation [3,4]. However, this embolic hypothesis does not fully match with all aspects of PAIS, such as: (*i*) the imbalance of PAIS distribution between the anterior vs. posterior intra-cranial arterial territories even taking into account the asymmetry of anterior vs. posterior blood flows; (*ii*) the infrequent occurrence of concomitant extra-cerebral infarcts; and (*iii*) the observations showing that 22%–40% of the largest multicentric cohorts of PAIS-affected infants assessed by magnetic resonance angiography presented abnormal vascular imaging compatible with arterial wall diseases [9,12]. These elements, along with the epidemiological association between PAIS, chorioamnionitis and umbilical cord vasculitis, give rise to the physiopathological hypothesis that an acute vasculitis specifically affects cerebral arteries supplying the cerebral territories vulnerable to PAIS [9,11]. To test this hypothesis, we used an original preclinical rat model of lipopolysaccharide (LPS)-induced chorioamnionitis previously designed in our laboratory [13]. Experiments performed in our laboratory on this model of LPS-induced materno-fetal inflammatory response showed that the interleukin-1 (IL-1) system, and especially the IL-1/IL-1 receptor antagonist (IL-1Ra) ratio played a key role in the pathophysiology of LPS-induced: (*i*) chorioamnionitis; (*ii*) macrophagic arteritis within the placenta and the umbilical cord; and (*iii*) fetal brain injuries [13]. This LPS-induced pro-inflammatory IL-1/IL-1Ra response was driven by activated macrophages [13]. These data support our hypothesis of a similar process occurring beyond the placental arteries, within the walls of intra-cranial arteries susceptible to PAIS.

## 2. Results

### 2.1. Constitutive Expression of Inflammatory Markers of Interest within the Medium-Sized Arterial Wall of Arteries Susceptible vs. Non-Susceptible to PAIS

**Figure 1:**
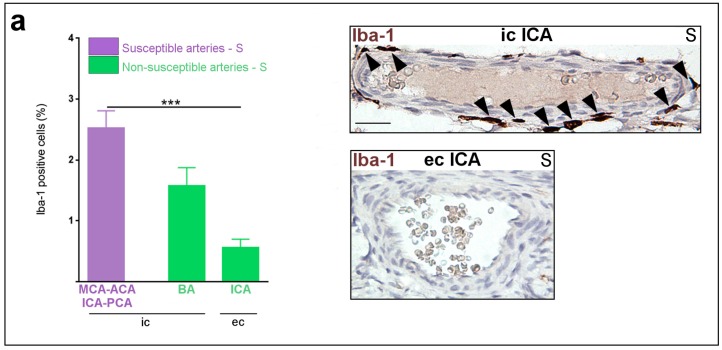

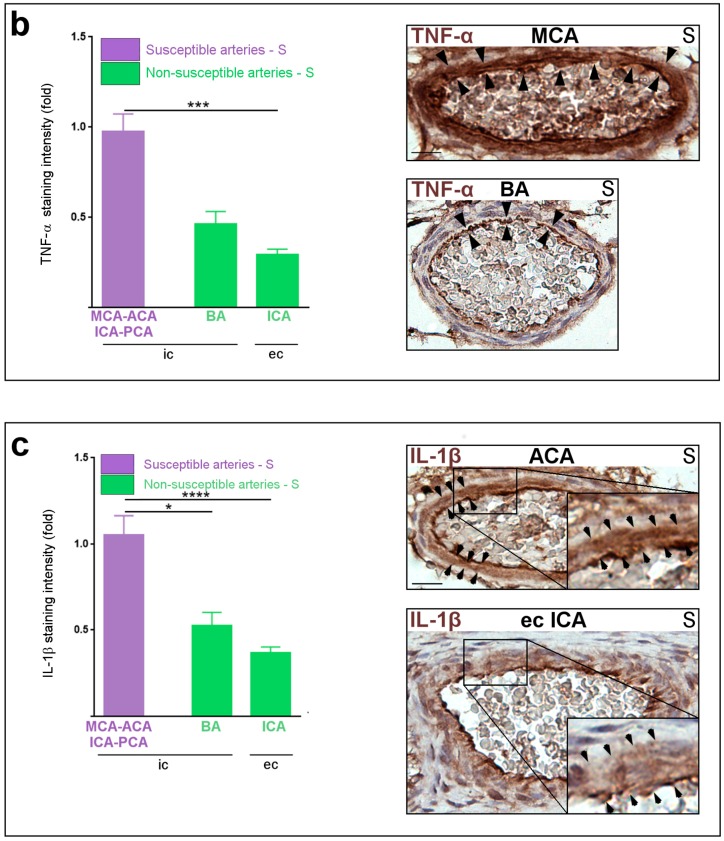
Macrophagic infiltration and pro-inflammatory cytokines expression within the arterial wall of PAIS-susceptible vs. non-susceptible artery of P1 pups from the S group: (**a**, **left** panel) Increased density of Iba-1+ cells in PAIS-susceptible vs. non-susceptible arteries; (**a**, **right** panels) Iba-1+ cells (black arrowhead) in a susceptible intra-cranial artery (icICA) vs. non-susceptible extra-cranial artery (ecICA) to PAIS; (**b**, **left** panel) increased expression of TNF-α in PAIS-susceptible vs. non-susceptible arteries; (**b**, **right** panels) increased TNF-α staining (black arrowheads) in PAIS-susceptible (MCA) vs. non-susceptible (BCA) arteries; (**c**, **left** panel) increased expression of IL-1β in PAIS-susceptible vs. non-susceptible arteries; and (**c**, **right** panels) increased IL-1β staining (black arrowheads) in PAIS-susceptible (ACA) vs. non-susceptible (ecICA) arteries. Data are presented as mean ± standard error of the mean (SEM). * *p* < 0.05, *** *p* < 0.001, **** *p* < 0.0001, Kruskal–Wallis test. Number (*n*) = 5–14 arteries from 5–7 animals per condition. Scale bar = 15 µm. Abbreviations: ACA, anterior cerebral artery; BA, basilar artery; ec, extra-cranial; Iba-1, Ionized calcium binding adapter molecule-1; ic, intra-cranial; ICA, internal carotid artery; IL-1β, Interleukin-1β; MCA, middle cerebral artery; PCA, posterior cerebral artery; S, saline; TNF-α, tumor necrosis factor-α.

**Figure 2:**
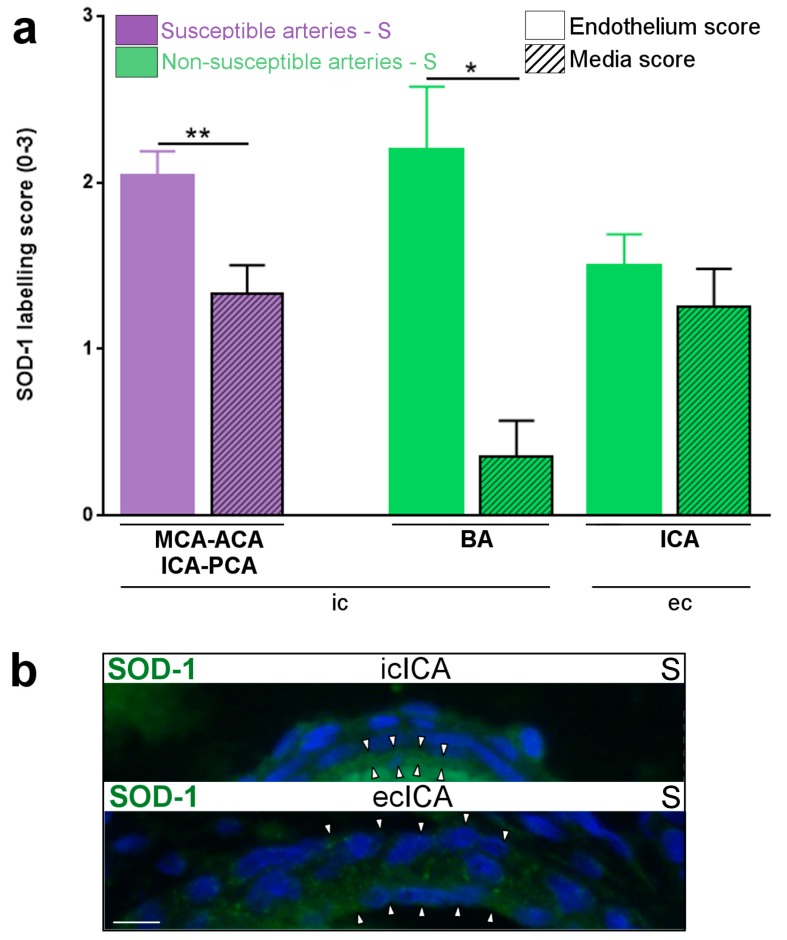
SOD-1 expression within the arterial wall of PAIS-susceptible vs. non-susceptible artery of P1 rat pups from the S group: (**a**) unbalanced expression of SOD-1 between intima and media of intra- but not extra-cranial arteries (labeling score; see method section and Figure S1); and (**b**) SOD-1 staining by IF (white arrowheads) in PAIS-susceptible (icICA) vs. non-susceptible (ecICA) arteries. Data are presented as mean ± standard error of the mean (SEM). * *p* < 0.05, ** *p* < 0.01, Mann–Whitney test. *n* = 5–9 arteries from 4–5 animals per condition. Scale bar = 7 µm. Abbreviations: ACA, anterior cerebral artery; BA, basilar artery; ec, extra-cranial; ic, intra-cranial; ICA, internal carotid artery; MCA, middle cerebral artery; PCA, posterior cerebral artery; S, saline; SOD-1, superoxide dismutase-1.

In pups from the S group at P1, a five-fold increased density of Iba-1+ macrophages was detected in the wall of medium-sized segments of intra-cranial arteries, including all those susceptible to PAIS, compared to ecICA (Figure 1). Most of these macrophages were detected within the adventitial layer (Figure 1a). TNF-α (Figure 1b) and IL-1β (Figure 1c) expressions were constitutively up-regulated in the wall (predominantly in intimal and medial layers) from medium-sized segments of arteries susceptible, vs. non-susceptible, to PAIS. The level of ROS (indirectly measured by SOD-1 expression) was lower in the media vs. intima from intra-cranial arteries, but was identical in both layers from extra-cranial arteries (Figure 2a,b).

### 2.2. Cytokines and Chemokines Involved in LPS-Induced Materno-Fetal Inflammatory Responses

**Figure 3:**
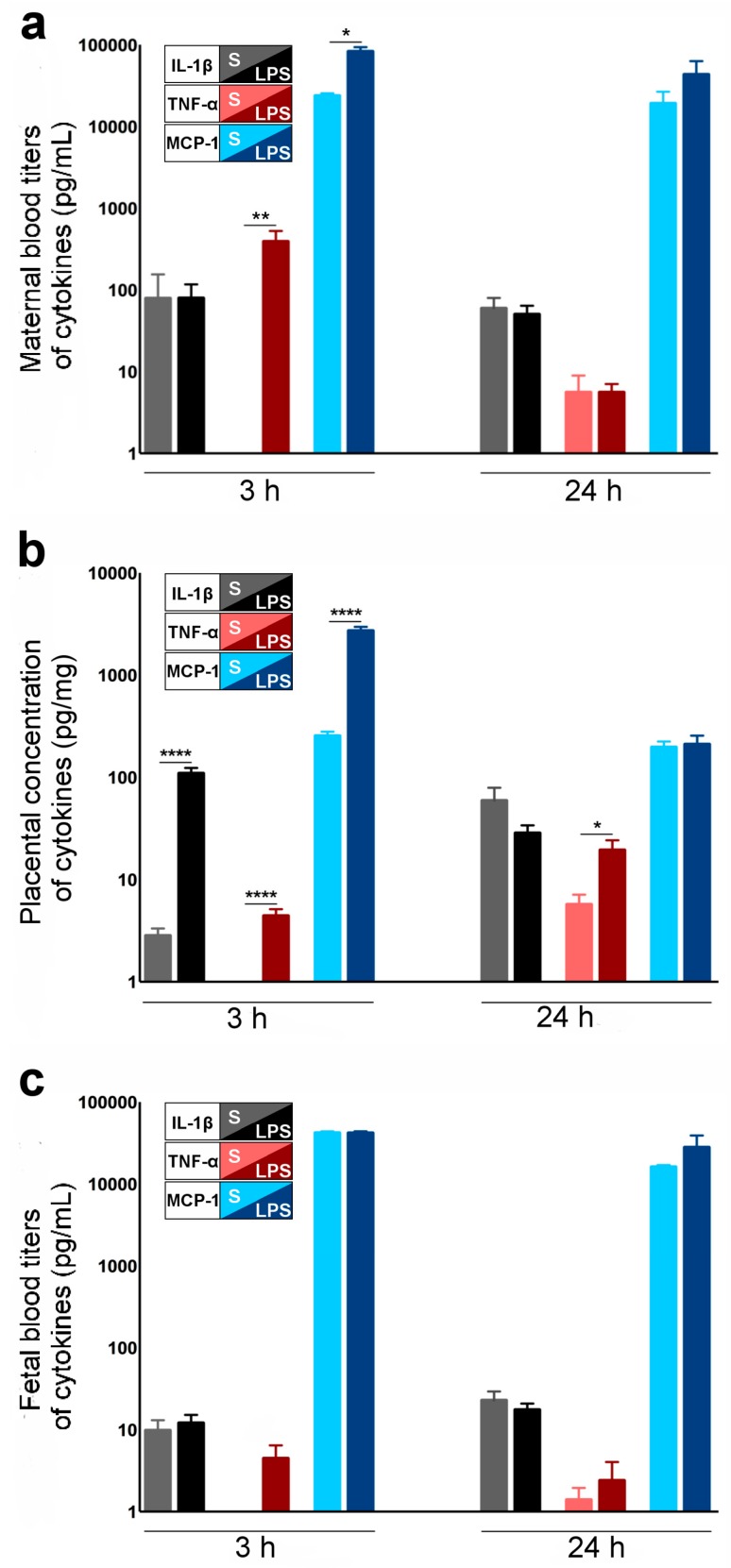
LPS-induced materno-fetal inflammatory response (LPS group vs. S group). (**a**) Increased TNF-α and MCP-1 blood titers of LPS- vs. S-exposed dams, at 3 h after the first injection of LPS; *n* = 3–4 dams in each experimental group; (**b**) Increased IL-1β, TNF-α, MCP-1 and TNF-α titers (ELISA) from LPS- (LPS group) vs. saline (S group)-exposed placentas (LPS group), at 3 h and 24 h after the initial exposure to LPS; *n* = 6–8 placentas in each experimental group; (**c**) Increased TNF-α fetal blood titers at 3 h after the first LPS injection; *n* = 6–8 fetuses in each experimental group. Data are presented as mean ± standard error of the mean (SEM). * *p* < 0.05, ** *p* < 0.01, **** *p* < 0.0001, Mann–Whitney test. Abbreviations: IL-1β, Interleukin-1β; LPS, lipopolysaccharide; MCP-1, monocyte chemotactic protein one; mL, milliliter; pg, picogram; TNF-α, tumor necrosis factor-α, S, saline.

TNF-α and MCP-1 (but not IL-1β) titers were up-regulated in the maternal blood 3 h after the first LPS injection (Figure 3a) (LPS group) compared to maternal blood from the S group. IL-1β, TNF-α and MCP-1 were up-regulated in the LPS- vs. S-exposed placentas at 3 h post-LPS injection (Figure 3b). At 24 h post-LPS, TNF-α remained increased in the placentas, whereas IL-1β and MCP-1 decreased back to the S group titers (Figure 3b). In the blood of LPS-exposed fetuses (LPS group), there was no increase of the inflammatory markers (TNF-α, IL-1β and MCP-1) at 3 h and 24 h after the initial exposure to LPS (Figure 3c) compared to S-exposed fetuses.

### 2.3. Susceptibility to Infarcts in the Pups from LPS-Exposed vs. S-Exposed Dams

**Figure 4:**
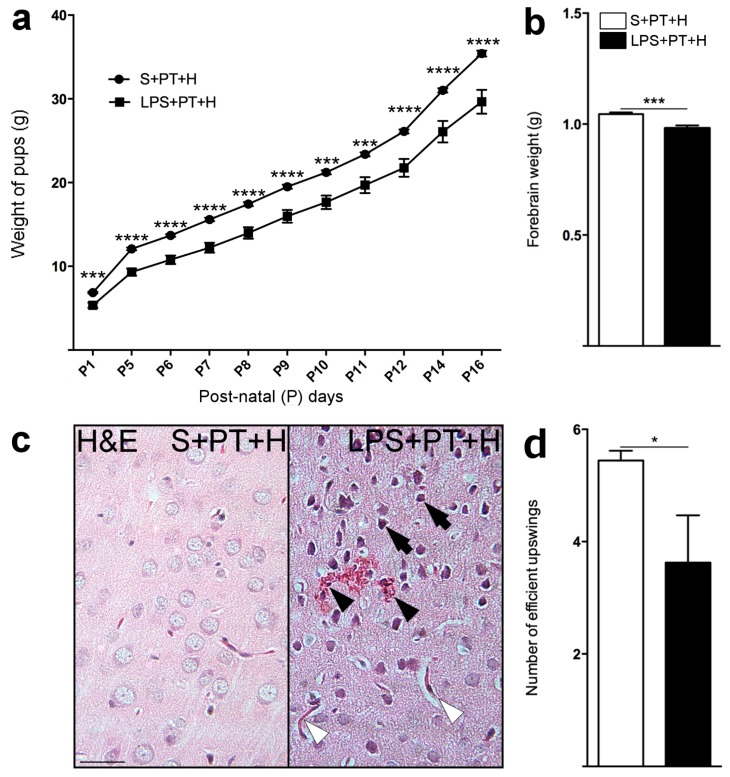
Outcome measures between pups from S + PT + H- vs. LPS + PT + H-group: (**a**) Default of body weight growth of pups exposed to LPS + PT + H vs. S + PT + H; (**b**) decreased weight of forebrains from P20 pups previously exposed to LPS + PT + H vs. S + PT + H; (**c**) infarcts featured by pyknotic neurons (black arrows), red blood cells infiltrates (black arrowheads), and neovessels (white arrowheads) in LPS + PT + H- but not in S + PT + H-condition; and (**d**) diminished number of efficient upswings of the LPS + PT + H vs. S + PT + H pups at P16. Data are presented as mean ± standard error of the mean (SEM). * *p* < 0.05, *** *p* < 0.001, **** *p* < 0.0001, Mann–Whitney test. *n* = 8–10 pups per experimental group. Scale bar = 30 µm. Abbreviations: g, gram; H, hypoxia; H&E, Hematoxylin & Eosin; LPS, lipopolysaccharide; P, postnatal day; PT, photothombosis; S, saline.

Pups from the LPS + PT + H group presented a significantly lower body weight (as measured from P1 to P16) compared to those from the S + PT + H group (Figure 4a). A mean 6% decrease of forebrain weight was observed in P20 pups from LPS + PT + H group vs. S + PT + H group (Figure 4b). Rats from LPS + PT + H group presented ischemic strokes featured by pyknotic neurons, spongiosis, neovascularization, loss of normal tissue cytoarchitecture and hemorrhagic infiltrates at P20 (Figure 4c) [14,15]. No stroke was detected in the pups from the S + PT + H group, whereas 62% of LPS + PT + H pups had at least one infarct (*p* < 0.01).

### 2.4. Motor Behavior of Pups Exposed to S + PT + H vs. LPS + PT + H

LPS + PT + H pups showed a significantly reduced number of efficient upswings compared to the S + PT + H pups (Figure 4d). Pups from LPS + PT + H group presented a significantly prolonged time (>1 s) to achieve a full righting reflex at P13 (*p* < 0.05) compared to pups from the S + PT + H group.

### 2.5. Inflammatory Responses within Cervicocephalic Arterial Walls of Pups in Utero-Exposed to LPS vs. S

**Figure 5:**
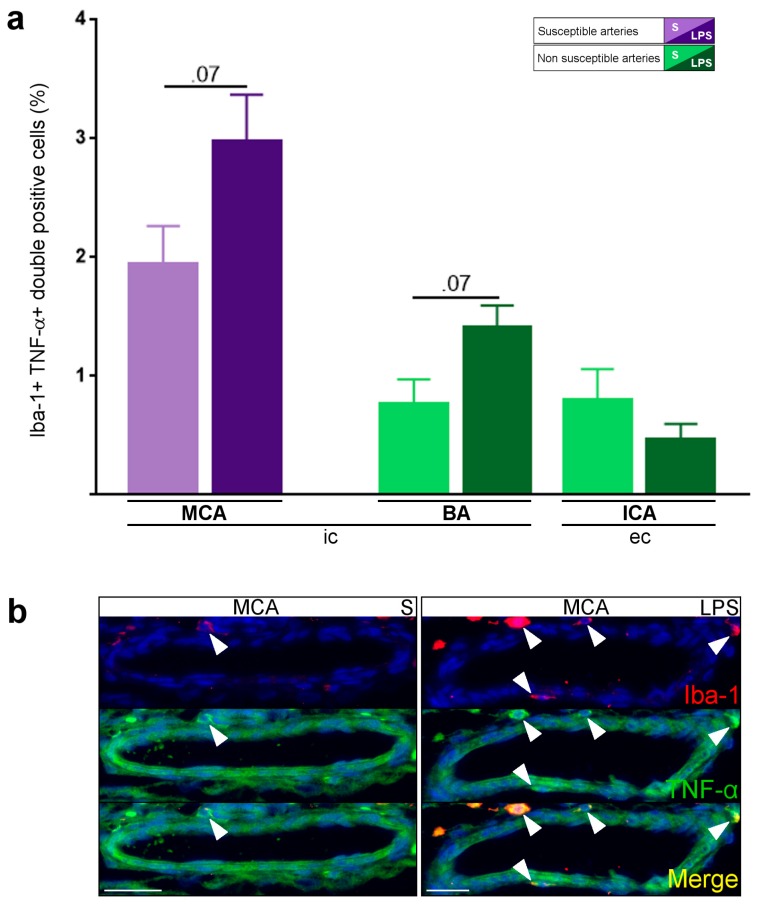
LPS-induced macrophagic arteritis (LPS group vs. S group) of intra- vs. extra-cranial arteries: (**a**) increased density of Iba-1+TNF-α+ cells in the walls of intra-cranial vs. extra-cranial arteries, in P1 pups in utero exposed to LPS vs. S; and (**b**) M1 phenotype of macrophages in the arterial wall (MCA) of LPS-exposed pups: Iba-1+TNF-α+ (M1, white arrowhead) double labeling. Data are presented as mean ± standard error of the mean (SEM). *p* = 0.07, Mann–Whitney test. *n* = 4–14 arteries from 4–7 animals per condition. Scale bar = 15 µm. Abbreviations: BA, basilar artery; ec, extra-cranial; Iba-1, Ionized calcium binding adapter molecule-1; ic, intra-cranial; ICA, internal carotid artery; LPS, lipopolysaccharide; MCA, middle cerebral artery; S, saline; TNF-α, tumor necrosis factor-α.

**Figure 6:**
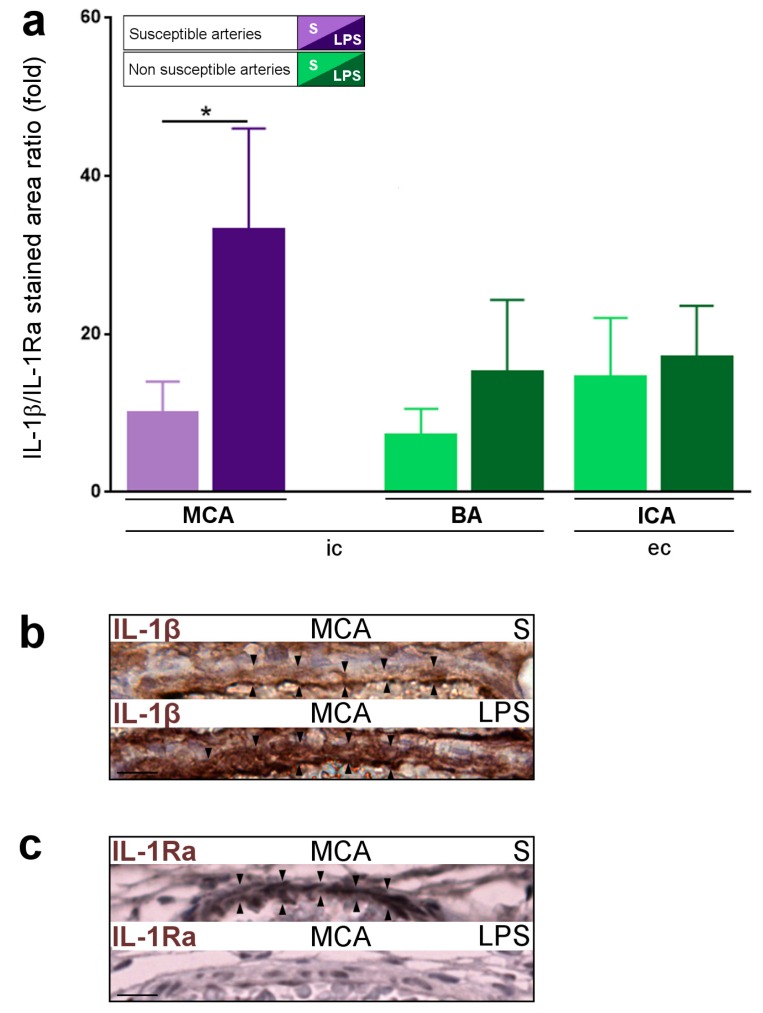
IL-1β/IL-1Ra pro-inflammatory ratio in LPS-exposed (LPS group) vs. S-exposed (S group) P1 pups: (**a**) increased IL-1β/IL-1Ra pro-inflammatory ratio in LPS-exposed (LPS group) vs. S-exposed (S group) PAIS-susceptible artery; (**b**) IL-1β staining (black arrowhead) in a LPS-exposed vs. S-exposed PAIS-susceptible artery (MCA); and (**c**) IL-1Ra staining (black arrowhead) in a LPS-exposed vs. S-exposed PAIS-susceptible artery (MCA). Data are presented as mean ± standard error of the mean (SEM). * *p* < 0.05, Mann–Whitney test. *n* = 6–14 arteries from 6–7 animals per condition. Scale bar = 15 µm. Abbreviations: BA, basilar artery; ec, extra-cranial; ic, intra-cranial; ICA, internal carotid artery; IL-1β, interleukin-1β; LPS, lipopolysaccharide; MCA, middle cerebral artery; S, saline.

The number of Iba-1+TNF-α+ pro-inflammatory macrophages (M1 phenotype) increased in the wall of the MCA from the LPS- vs. S-group (Figure 5a,b). In contrast, the number of Iba-1+TNF-α+ M1 macrophages did not change in the wall of extra-cranial PAIS-non-susceptible arteries from the LPS vs. S group (Figure 5a). There was no CD3 lymphocyte or PMN cell detected in the arterial walls of P1 pups from both S and LPS groups. The IL-1β/IL-1Ra pro-inflammatory ratio was significantly increased in the wall of the MCA from the LPS compared to the S-group (Figure 6a–c). In contrast, the IL-1β/IL-1Ra pro-inflammatory ratio remained unchanged in the wall of PAIS-non-susceptible arteries from the LPS vs. S group (Figure 6a). There was no difference of expression of ICAM-1 (Figure S2a), P-selectin (Figure S2b), SOD-1 (Figure S2c,d), and tissue factor (Figure S2e) between arterial walls from S- vs. LPS-group from P1 pups. Macrophages (Iba-1+) from the arterial wall of PAIS-susceptible and non-susceptible arteries were negative for PCNA. There was no expression of CD40/CD40L, vWF, and iNOS within the arterial wall or at the surface of endothelial cells in P1 pups from S- vs. LPS-group (data not shown).

## 3. Discussion

Constitutively, we observed a higher adventitial density of macrophages, a higher intimal vs. medial expression of ROS, and higher expressions of both TNF-α and IL-1β within the wall of intra- compared to extra-cranial arteries. This could be correlated to the specific vulnerability to vasculopathies of intra- vs. extra-cranial arterial wall not only in human neonates but also later on in childhood in diseases such as moyamoya or transient (focal) cerebral arteriopathies [1,16,17,18]. This distinctive phenotype between intra- vs. extra-cranial arteries might be linked to differential neural crest cell contribution that is more important in arteries embryologically arising from icICA (namely, ACA, MCA, and PCA) than others [19].

Our results also show that *in utero* exposure to LPS led to a materno-fetal inflammatory response implicating IL-1β, TNF-α, and MCP-1. This LPS-induced materno-fetal inflammatory response was associated to a neonatal cerebral arteritis involving M1 macrophages increased density and pro-inflammatory orientation of the IL-1 system within the wall of intra-cranial arteries susceptible to PAIS. Finally, a subthrombotic state triggered infarcts within the MCA territory of pups *in utero*-exposed to LPS (LPS + PT + H group); this effect was not observed in S-exposed pups (S + PT + H group). 

**Figure 7:**
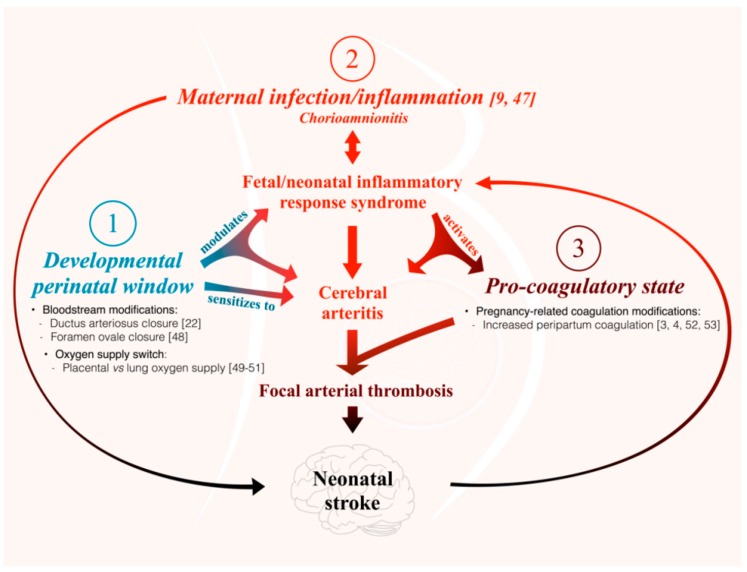
Physiopathological paradigm of simultaneously combined perinatal triple risk factors leading to PAIS.

To reduce the cerebral blood flow, this intra-cerebral arteritis might either trigger a thrombotic process in the lumen of the inflamed arterial wall, or a vasospasm, or both. The physiological perinatal prothrombotic state, and the pathogen-induced up-regulation of materno-fetal inflammatory markers, could contribute to such clot formation in the lumen of inflamed arteries. We did not detect a LPS induced increase of tissue factor at the endothelial surface of PAIS-susceptible arteries. This suggests that other thrombotic factor(s) might be involved in triggering the thrombotic cascade leading to PAIS. Following LPS-induced materno-fetal immune activation, TNF-α and IL-1β were over-expressed in the placenta and within the arterial walls susceptible to PAIS suggesting that blood platelets might well be activated through the pro-coagulant effect of these pro-inflammatory cytokines [20]. The higher expression of ROS in the endothelium of intra-cranial arteries susceptible to PAIS could also contribute to platelet activation [21]. Besides, a focal vasospasm, as involved in the neonatal pO_2_/ROS-induced ductus arteriosus remodeling and in some vasculitic processes, might also occur [22]. Pro-inflammatory cytokines could likewise contribute to vasospasm, e.g., via the up-regulation of endothelin-1 [23]. Altogether—as summarized in Figure 7—preclinical, human clinical and neuropathological data suggest that focal vasculitis, prothrombotic activation and vasospasm act in concert in the arterial wall, and within the lumen of cerebral arteries susceptible to PAIS [9,11]. The subsequent disruption of the arterial blood flow might affect sensitized neurons through the release of pathogen-induced microglial soluble factors acting directly on neural cells to exacerbate their sensitivity to hypoxia [24].

This observational study has several limitations:

(i) The present study is the first to our knowledge to address the question of the differential susceptibility to stroke of the main cerebral arteries using an animal model. We are not aware of any previous works documenting the distribution of PAIS in rodents. However, previous experiments performed in our laboratory on this rat model of LPS-induced materno-fetal inflammatory response showed that fetuses from LPS-exposed dams developed macrophagic arteritis within their placenta and umbilical cord [13]. This observation led us to hypothesize that such macrophagic arteritis affecting the umbilical cord might propagate to other arteries, such as brain arteries. Based on these previous findings, we assumed that rat could be a pertinent animal model to deal with this research question and to study the biological mechanisms underpinning the various susceptibilities to stroke.
(ii) Our study is based on semi-quantitative IHC due to the difficulty in extracting a sufficient amount of proteins from tiny cerebral arteries of rodents to make possible the use of some classic quantitative analyses. Selective dissections of such fragile arteries would have necessitated strenuous and lengthy dissections of the skull to access the small basal arteries. Such arterial isolation without contamination by other tissues (e.g., meningeal tissue) and without alterations due to too long delay and trauma remained an unresolved challenge in our hands. Further experiments on bigger animals might overcome this limitation. Due to the small amount of cerebrospinal fluid (CSF) and its difficult access in sufficient amounts in small animals, it was not possible to monitor CSF inflammation. It is possible that the adventitial macrophagic activation we observed in segments of arteries located within or in the vicinity of subarachnoidal spaces would result from LPS-induced CSF inflammation.
(iii) An important question in relation to our model is whether LPS crosses the placental barrier. According to the literature, the effects of maternal LPS exposure on the developing fetus are not mediated directly by the LPS [25,26] but via an indirect effect that might implicate the fetal immune response [13,27,28,29].
(iv) This study used newborn rat pups whose brain development corresponds to a stage of development equivalent to preterm human newborns whereas PAIS occurs in term human newborns. However, there is no data to our knowledge showing that the cerebral arteries of newborn rats are less mature than those of term human newborns. Our aim was to study the *short-term* interaction between end-gestational/placental inflammation and perinatal cervicocephalic arteries. This would not be feasible in rodents at P12, i.e., at a stage of brain development equivalent to term human newborns. Given that placental inflammation (chorioamnionitis) is a major risk factor of PAIS, and that PAIS most often occurs just a few hours after birth, designing our model in P1 newborn rats appeared to be relevant to the reality of this pathology.
(v) Additional pre-clinical, as well as clinical studies, for instance using non-invasive neonatal arterial wall magnetic resonance imaging, are mandatory to confirm our preclinical findings, their translation to human, and thus, to further uncover the pathophysiological mechanisms underlying human PAIS.

## 4. Materials and Methods

### 4.1. Animals

**Figure 8:**
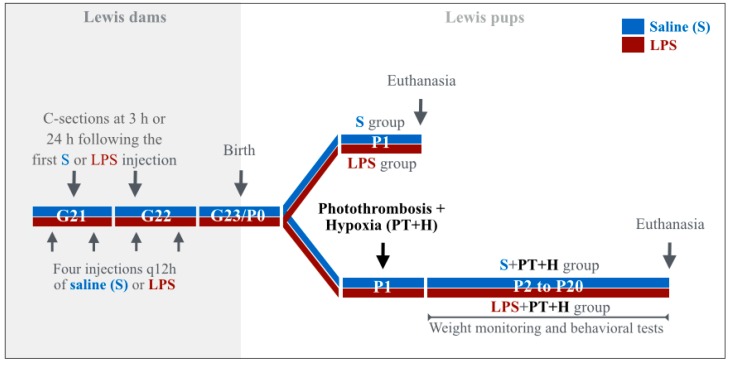
Summary of the experimental designs used for each animal subgroup. Abbreviations: G, gestational day; H, hypoxia; LPS, lipopolysaccharide; P, perinatal; PT, photothrombosis; q, every (from Latin *quaque*); S, saline.

Animal experiments were conducted as previously described [30]. Pregnant primiparous Lewis rats were obtained from Charles River Laboratories (Saint-Constant, QC, Canada). They were individually housed in standard polypropylene opaque cages (47.0 × 25.0 × 14.5 cm) with filtered lids in a quiet and controlled room in our animal facility starting on gestational day (G) 13. They were reared on a 12 h light/dark cycle (6 a.m.–6 p.m.) at 20–23 °C with access to sterilized laboratory chow (Charles River Laboratories) and water ad libitum. Dams were randomized into two groups: they were either injected intra-peritoneally (ip) between gestational day (G) 21 and G 22 with lipopolysaccharide (LPS, 0127:B8, Sigma, Oakville, ON, Canada) from *Escherichia coli* (LPS group) at a dose of 60 μg/12 h (h), or with a pyrogen-free saline (saline (S) group) as presented in Figure 8. Adjusted to the dams’ weight, the injected dose of LPS was 240 ± 30 μg/kg/12 h. All dams had four injections of S or LPS except those undergoing cesarean section after the first (3 h following the first injection) or second S- or LPS-injection (24 h following the first injection) as explained in Figure 8. The dams were observed every 12 h after injection to detect sickness-related behaviors (fever, pain, grooming or hypoactivity). Our objective was to replicate as far as possible a pre-natal end-gestational infectious/inflammatory aggression, as observed in human and gestational chorioamnionitis often due to *Escherichia coli*. Placenta and fetuses from a subgroup of dams were extracted by caesarean section performed at G21 (3 h post-S or LPS injection) and G22 (24 h post-S or LPS injection), as previously described [13] (Figure 8). The timing of caesarean section and subsequent prenatal and postnatal sampling in S and LPS groups was chosen according to the expected delay of LPS-induced release of inflammatory molecules of interest involved in the materno-fetal inflammatory response syndrome, as previously shown in other similar preclinical models from our laboratory of LPS-induced prenatal neuroinflammation [13,14]. Other subgroups of S or LPS-exposed dams gave birth naturally; part of the offspring was euthanized at postnatal day (P) 1 by decapitation to collect the heads in order to study the S vs. LPS-exposed arteries of interest (Figure 8). Given that PAIS occurs mostly during the first 24 h after birth, we chose this P1 time point. The remaining P1 offspring from S or LPS-exposed dams was submitted to a prothrombotic stress: transcutaneous laser photothrombosis (PT; laser intensity: 125 mW for 2 min (min)) applied on both MCA segments located between the ear and the eye [31] following aluminium phthalocyanine tetrasulfonate (AlPcS_4_) ip exposure. AlPcS_4_ (650 µg/kg) is a non-toxic photosensitizer used in animal models [32], which was injected ip 30 min before laser illumination [33]. Due to the presence of oxygen within the blood, AlPcS_4_ produces free cytotoxic radicals when exposed to transcutaneous laser light. This creates a focal intra-luminal sub-thrombotic effect [34]. Pups were submitted 45 min later to hypoxia (H) for 3 h 30 min (8% O_2_) as previously described [35] (Figure 8). Exposure to H is a classic mean used to minimize the capacity of neonatal rat arteries to compensate for the impact of reduced cerebral arterial blood flow through the circle of Willis anastomoses [15]. Pups were weighed from P1–P16 and euthanized at P20, as previously described [36]. The brains at P20 were collected and weighed after removal of olfactory bulbs and cerebellum. This P20 time point was chosen to detect fully established brain infarcts.

In summary (Figure 8), four experimental groups were studied: (1) S group (S only-exposed dams, fetuses, and pups); (2) LPS group (LPS only-exposed dams, fetuses, and pups); (3) S + PT + H group (pups in utero exposed to S and post-natally to PT + H); and (4) LPS + PT + H group (pups in utero exposed to LPS and post-natally to PT + H). 

We used 29 dams and 196 fetuses or pups. Samples of maternal blood, fetal blood and placentas were collected and stored at −80 °C. Samples of P1 heads and P20 brains were fixed in paraformaldehyde 4% and embedded in paraffin as previously described [30,36]. 

The animal protocol used in this work was evaluated and approved by the Institutional Animal Care Committee of the Université de Sherbrooke (Protocol 147-11R) in accordance with the Canadian Council on Animal Care (CCAC) guidelines.

### 4.2. Immunohistochemistry (IHC), Immunofluorescence (IF)

Previous data from our laboratory showed that the interleukin-1 (IL-1) system, and especially the IL-1/IL-1Ra ratio, plays a key role in the pathophysiology of LPS-induced macrophagic placental vasculitis [13,30]. These results prompted us to test the hypothesis of a similar process occurring beyond the placental arteries, within the walls of intra-cranial arteries susceptible to PAIS implicating the above-mentioned molecules, and related molecular cascade involving adhesion molecules and chemokines, such as: monocyte chemoattractant protein-1 (MCP-1), cluster of differentiation 40 (CD40), cluster of differentiation 40 ligand (CD40L), intercellular adhesion molecule-1 (ICAM-1), and/or proliferation (proliferating cell nuclear antigen (PCNA), free radical release (superoxide dismutase-1 (SOD-1)), and associated thrombotic molecules (tissue factor, vonWillebrand factor (vWF)).

Rat pups were euthanized by decapitation at P1. Heads of P1 pups were harvested and paraffin-embedded. IHC and IF were performed as previously described [30]. Consecutive heads’ sections were incubated with different set of primary antibodies and the corresponding secondary antibodies: ionized calcium binding adapter molecule-1 (Iba-1, 1:250, Wako, 019-19741, Richmond, VA, USA), IL-1β (1:50, AbD Serotec, AAR15G, Raleigh, NC, USA), IL-1Ra (1:25, Santa Cruz, sc-25444, Dallas, TX, USA), TNF-α (1:50, Millipore, AB1837P, Billerica, MA, USA), inducible NO synthase (iNOS, 1:50, Santa Cruz, sc-650G, Dallas, TX, USA) and arginase-1 (Arg-1, 1:20, Santa Cruz, sc-20150, Dallas, TX, USA)—to determine the pro-inflammatory (Iba-1+TNF-α+ M1 phenotype) vs. anti-inflammatory (Iba-1+Arg-1+ M2 phenotype) of macrophages [37]. Cluster of differentiation 3 (CD3, 1:50, Abcam, ab5690, Toronto, ON, Canada), MCP-1 (1:40, Millipore, AB1834P, Billerica, MA, USA), CD40 (1:100, Santa Cruz, sc-975, Dallas, TX, USA), CD40L (1:250, Abcam, ab65854, Toronto, ON, Canada), ICAM-1 (1:20, Santa Cruz, sc-8439, Dallas, TX, USA), PCNA (1:500, Santa Cruz, sc-56, Dallas, TX, USA), tissue factor (1:250, Abcam, ab104513, Toronto, ON, Canada), vWF (1:40, LS Bio, LS-B4034/48362, Seattle, WA, USA), polymorphonuclear neutrophils (PMN, 1:250; Cedarlane, CALD51140, Burlington, ON, Canada), and SOD-1 (1:100, Abcam, ab13498, Toronto, ON, Canada) were used to define other inflammatory and coagulatory features of cerebral arteries [38].

We divided the rat arteries in two groups based on their susceptibility to PAIS in human [1,25], namely the susceptible group including ACA, MCA, icICA and PCA (all embryologically arising from the embryonic icICA [7]) vs. the non-susceptible group including ecICA and BA, given that territories of ecICA and BA (including ophthalmic and brainstem territories) are not affected by PAIS. Each staining was performed in one to six section(s) per segment of interest from each artery, corresponding to Bregma 0.20 mm to −2.30 mm for ACA, MCA and icICA, or −6.72 mm to −8.72 mm for PCA, ecICA and BA [39,40]. Further experiments targeted the MCA, which is the main artery vulnerable to PAIS [9,12]. Labeling was assessed by conventional and confocal microscopies. We used three different techniques for the IHC and IF staining assessments, as previously described [36,41,42,43]: (*i*) for some IHC (Iba-1, ICAM-1, P-selectin, PMN, CD3, CD40, CD40L, and vWF) and IF (Iba-1, TNF-α, IL-1β PCNA, iNOS, and Arg-1) labeling, counting of labeled and unlabeled cells (data presented as ratio between labeled and total cells (%)) was performed using a positive vs. negative score; (*ii*) for the SOD-1 labeling by IF, we used a 0–3 score, in which 0 = no labeling, 1 = low-intensity, 2 = mid-intensity, and 3 = high-intensity labeling (Figure S1, data presented using this labeling score); and (*iii*) for other IHC staining assessment (IL-1β, IL1-Ra, TNF-α, and tissue factor), we used the ImageJ analysis software [44] (data presented as staining intensity and/or stained area). We first separated the immunostaining (brown) from the hematoxylin stain (purple) using the extract-brown plugin generated by the image analysis platform of the Centre de Recherche du Centre Hospitalier Universitaire de Sherbrooke (PAVI: plateforme d’analyse et de visualisation d’images). This plug-in is a software component adding a specific ability to ImageJ software that permits the distinction between the different colors and the brown generated by the 3,3-diaminobenzidine (DAB)-specific staining. We then set arbitrary staining thresholds that were specific to each marker from representative saline-exposed arteries. These threshold levels were set to detect the highest immunostaining intensity and then applied to all other samples, allowing quantitative comparisons of staining levels. One person blinded to the identity of the experimental groups performed the quantification. However, as the arteries are easily recognizable according to their anatomical position, it was not possible for the experimenter to be blinded for their names and therefore their categories: perinatal arterial ischemic stroke (PAIS) susceptible vs. non-susceptible arteries. All these techniques are routinely performed in our laboratory [41,43]. A NanoZoomer (Hamamatsu Corporation, Bridgewater, NJ, USA) device was used. Figures were created using GraphPad Prism (GraphPad Software Inc., La Jolla, CA, USA) and GNU Image Manipulation Program [45].

### 4.3. ELISA

Placental proteins were extracted and concentrations determined, as previously described [30]. Plasma samples were obtained by centrifugation of whole blood (15 min, 13,000 RPM) in plasma separator tubes with lithium heparin (Becton Dickinson, Franklin Lakes, NJ, USA). All the samples were kept at −80 °C until their analysis. Cytokines (IL-1β, TNF-α) and chemokine (MCP-1) were quantified using ELISA kits according to manufacturer’s instruction (R&D System, Minneapolis, MN, USA; except for MCP-1: Becton Dickinson, Franklin Lake, NJ, USA).

### 4.4. Behavioral Tests

Motor behavior was assessed at P13 and P16. Righting reflex was tested at P13. Rats were placed on their back, four paws in the air. The time to move from paws in the air to four paws on the ground was measured and the pups were classified as having an impaired righting reflex when the delay to achieve a full righting reflex was more than 1 s. In the Elevated Body Swing Test (EBST) P16 rats were held by the base of the tail head downwards during 20 s, as previously described [42,46]. The number of effective upswings (+90°) and the total number of upswing were compared between the S + PT + H and LPS + PT + H groups. A decrease in the number of effective upswings or in the total number of upswings during 20 s was considered as a motor deficit.

### 4.5. Statistical Analyses

Data are presented as mean ± standard error of the mean (SEM). Given that neonatal AIS occur most often unilaterally, right and left arterial trees were considered as independent entities [1,47]. For all data, averages were extracted from different sections of the arteries of interest and this value was used for the statistical analysis. Comparisons between groups were performed using the Mann–Whitney test, the Kruskal–Wallis test or the Fisher’s exact test. The GraphPad outlier calculator (available online: http://www.graphpad.com/quickcalcs/Grubbs1.cfm) was used to detect and remove outlier values. The significance level was set at *p* < 0.05. All the graphs were created using the GraphPad Prism 7 software. The artwork was performed using the GNU Image Manipulation Program [45]. The number of animals used in the experiments was mentioned in each figure legends.

## 5. Conclusions

The observations made on this preclinical model lead us to propose a physiopathological paradigm whereby PAIS derives from a triple risk combination: (*i*) a materno-fetal inflammation leading to focal intra-cranial arteritis affecting selectively arteries arising from the carotid tree [9,47]; (*ii*) a developmental window of susceptibility [22,48,49,50,51]; and (*iii*) a physiological perinatal pro-coagulatory state reinforced by the materno-fetal inflammatory response [3,4,52,53] (Figure 7).

## Supplementary Materials

Supplementary materials can be found at www.mdpi.com/1422-0067/17/12/1980/s1.

## Abbreviations

ACA	Anterior cerebral artery
AlPcS_4_	Aluminium phthalocyanine tetrasulfonate
Arg-1	Arginase-1
BA	Basilar artery
CD40	Cluster of differentiation 40
CD40L	Cluster of differentiation 40 ligand
CP	Cerebral palsy
CSF	Cerebrospinal fluid
DAB	3,3-diaminobenzidine
EBST	Elevated Body Swing Test
ec	Extra-cranial
ELISA	Enzyme-linked immunosorbent assay
G	Gestational day
GIMP	GNU Image Manipulation Program
H	Hypoxia
Iba-1	Ionized calcium binding adapter molecule-1
ICAM-1	Intercellular adhesion molecule-1
ic	Intra-cranial
ICA	Internal carotid artery
IF	Immunofluorescence
IHC	Immunohistochemistry
IL-1β	Interleukin-1β
IL-1Ra	IL-1 receptor antagonist
iNOS	Inducible nitric oxide synthase
ip	Intra-peritoneally
LPS	Lipopolysaccharide
MCA	Middle cerebral artery
MCP-1	Monocyte chemotactic protein 1
mW	milliWatt
NO	Nitric oxide
P	Post-natal day
PAIS	Perinatal arterial ischemic stroke
PCA	Posterior cerebral artery
PCNA	Proliferating cell nuclear antigen
PMN	Polymorphonuclear neutrophils
PT	Photothrombosis
S	Saline
SEM	Standard error of the mean
SOD-1	Superoxide dismutase-1
TNF-α	Tumor necrosis factor-α
vWF	vonWillebrand factor
