# Supplementary material for: Perinatal Arterial Ischemic Stroke Is Associated to Materno-Fetal Immune Activation and Intracranial Arteritis
Source: Int J Mol Sci. 2016 Nov 25;17(12):1980. doi: 10.3390/ijms17121980 (PMC5187780; doi:10.3390/ijms17121980)
Supplement: Supplementary file 1 [file ijms-17-01980-s001.pdf]

Supplementary Materials: Perinatal Arterial Ischemic Stroke Is Associated to Materno-Fetal Immune Activation and Intracranial Arteritis

Clémence Guiraut, Nicole Cauchon, Martin Lepage and Guillaume Sébire

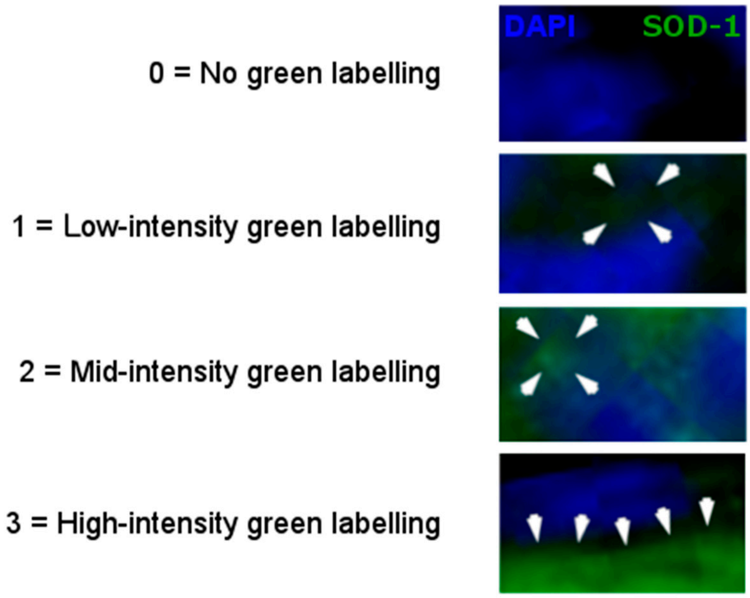

Figure S1. Illustration of our labeling score according to the level of expression of SOD-1 in the arterial walls of interest. This score was used to assess the SOD-1 labeling intensity on a 0–3 scale. White arrowheads indicate the SOD-1 labeling intensities corresponding to levels 1, 2 or 3 in our scale. Abbreviation: SOD-1, superoxide dismutase 1.

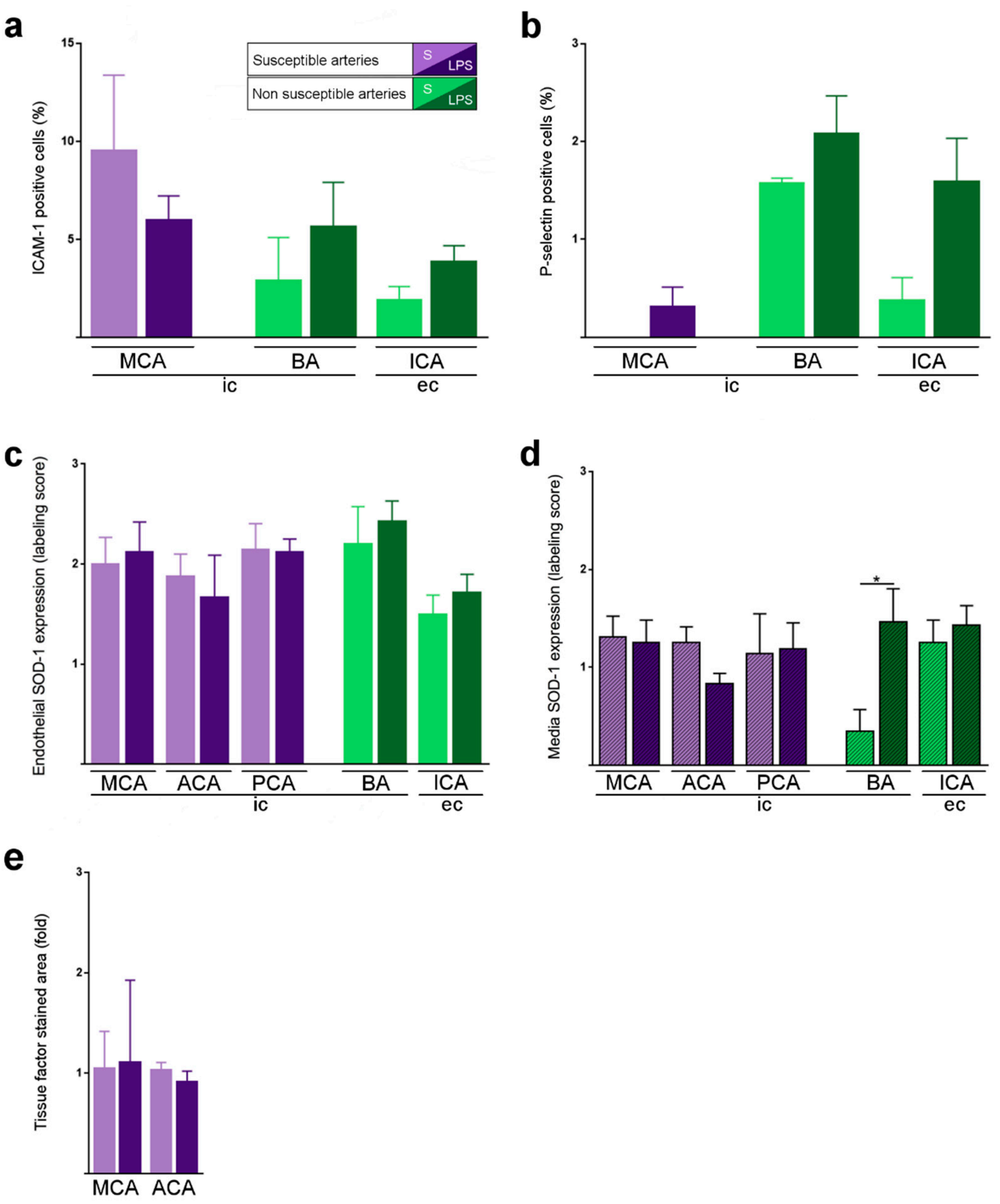

Figure S2. ICAM-1, p-selectin, SOD-1, and tissue factor expressions within the arterial walls of LPS-exposed (LPS group) vs. S-exposed (S group) P1 pups. (**a**) Similar number of ICAM-1+ cells detected in the walls of arteries susceptible vs. non-susceptible to PAIS, in P1 pups in utero exposed to LPS vs. S; (**b**) Similar number of p-selectin+ cells in the walls of arteries susceptible vs. non-susceptible to PAIS, in P1 pups in utero exposed to LPS vs. S; (**c**,**d**) Labeling score showing similar expressions of SOD-1 within the intima (**c**) and the media (**d**) of PAIS-susceptible vs. non-susceptible arteries from LPS-exposed vs. S-exposed P1 pups; (**e**) Similar expression of tissue factor in the walls of arteries susceptible to PAIS, in P1 pups in utero exposed to LPS vs. S. * $p < 0.05$, Mann-Whitney test; (**a**–**d**) Number (n) = 3–8 arteries from 3–4 animals per condition; (**e**) Number (n) = 3 arteries from 2 animals per condition. Abbreviations: ACA, anterior cerebral artery; BA, basilar artery; ec, extracranial; ic, intracranial; ICA, internal carotid artery; ICAM-1, intercellular adhesion molecule 1; LPS, lipopolysaccharide; MCA, middle cerebral artery; PCA, posterior cerebral artery; S, saline; SOD-1, superoxide dismutase-1; TNF-α, tumor necrosis factor-α.
